# Apigenin Impacts the Growth of the Gut Microbiota and Alters the Gene Expression of *Enterococcus*

**DOI:** 10.3390/molecules22081292

**Published:** 2017-08-03

**Authors:** Minqian Wang, Jenni Firrman, Liqing Zhang, Gustavo Arango-Argoty, Peggy Tomasula, LinShu Liu, Weidong Xiao, Kit Yam

**Affiliations:** 1 Dairy and Functional Foods Research Unit, Eastern Regional Research Center, Agricultural Research Service, US Department of Agriculture, 600 E Mermaid Lane, Wyndmoor, PA 19038, USA; Minqian.Wang@ars.usda.gov (M.W.); peggy.tomasula@ars.usda.gov (P.T.); linshu.liu@ars.usda.gov (L.L.); 2 Department of Food Science, Rutgers University, 65 Dudley Road, New Brunswick, NJ 08901, USA; kyam@sebs.rutgers.edu; 3 Department of Computer Science, Virginia Tech, 114 MCB Hall, Blacksburg, VA 24060, USA; lqzhang@cs.vt.edu (L.Z.); gustavo1@vt.edu (G.A.-A.); 4 Department of Microbiology and Immunology, Temple University School of Medicine, 3400 North Broad Street, Philadelphia, PA 19140, USA; WXiao@temple.edu

**Keywords:** apigenin, *Enterococcus*, gut microbiota, single molecule RNA sequencing

## Abstract

Apigenin is a major dietary flavonoid with many bioactivities, widely distributed in plants. Apigenin reaches the colon region intact and interacts there with the human gut microbiota, however there is little research on how apigenin affects the gut bacteria. This study investigated the effect of pure apigenin on human gut bacteria, at both the single strain and community levels. The effect of apigenin on the single gut bacteria strains *Bacteroides galacturonicus*, *Bifidobacterium catenulatum*, *Lactobacillus rhamnosus* GG, and *Enterococcus caccae*, was examined by measuring their anaerobic growth profiles. The effect of apigenin on a gut microbiota community was studied by culturing a fecal inoculum under in vitro conditions simulating the human ascending colon. 16S rRNA gene sequencing and GC-MS analysis quantified changes in the community structure. Single molecule RNA sequencing was used to reveal the response of *Enterococcus caccae* to apigenin. *Enterococcus caccae* was effectively inhibited by apigenin when cultured alone, however, the genus *Enterococcus* was enhanced when tested in a community setting. Single molecule RNA sequencing found that *Enterococcus caccae* responded to apigenin by up-regulating genes involved in DNA repair, stress response, cell wall synthesis, and protein folding. Taken together, these results demonstrate that apigenin affects both the growth and gene expression of *Enterococcus caccae*.

## 1. Introduction

**Figure 1:**
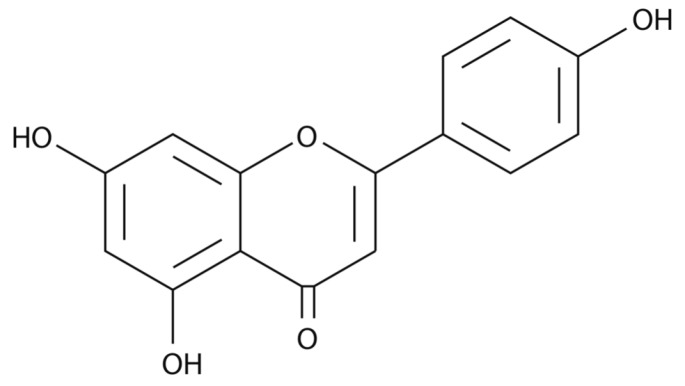
The chemical structure of apigenin (5,7-dihydroxy-2-(4-hydroxyphenyl)-4*H*-1-benzopyran-4-one).

The polyphenol apigenin (Figure 1) is one of the major flavonoids found in many fruits, vegetables, and herbs. Celery and parsley in particular provide high levels of apigenin, approximately 19 and 215 mg per 100 g, respectively [1], but it is also found in rutabagas, oranges, onions, wheat sprouts, tea, cilantro, and chamomile [2]. Daily intake varies with diet and geographical location, making it difficult to calculate an average intake for a large population. One study in The Netherlands determined the average daily intake of major flavones, such as apigenin, to be about 16 mg per day [3], whereas the average daily intake of apigenin was about 1 mg in a Dutch diet [4] and 1.5 ± 4.9 mg/day (range 0–30.3) in a group of female Flemish dietitians [5].

Research has found that a diet rich in the flavonoid apigenin has multiple beneficial properties. Apigenin has been identified as an active ingredient in the traditional, antibacterial, herbal medicines *Scutellaria barbata D.* Don (Lamiaceae) [6], *Castanea sativa* Mill. (Fagaceae) [7], *Portulaca oleracea* L. [8], *Marrubium globosum* ssp. *Libanoticum* [9], *Combretum erythrophyllum* (Combretaceae) [10], and *Aquilegia oxysepala* [11]. These herbal medicines have been used in treating multiple symptoms including coughing [7], digestive and biliary complaints [9], abdominal pains and venereal diseases [10], gynopathy, irregular menstruation and metrorrhagia [11] in different cultures. Studies using eukaryotic cells have found apigenin to be protective against multiple types of cancer [2], including colon cancer, and therefore apigenin is considered a promising plant-derived chemopreventive agent [12]. In eukaryotic cells, apigenin exhibits anti-inflammatory properties [13] and is considered an antioxidant, protecting DNA against free radicals generated by H_2_O_2_ or Fe^2+^ [14].

Apigenin is a flavonoid, which is a type of plant polyphenol. It is present in food and ingested as its glycoside conjugates, primarily as apigenin-7-*O*-glucoside, and acylated derivatives [15]. These forms are water soluble, and their structures have a major impact on their absorption and bioavailability, with apigenin bound to β-glycosides having the best bioavailability [12]. About 5–10% of total polyphenol intake, mostly monomers and dimers, are absorbed in the small intestine [16]. Since only a small portion is absorbed, it is estimated that 90–95% of total polyphenols ingested reach the colon region intact [16]. One study done on rats detected that 28.6% of ingested apigenin was excreted in feces within 72 h after oral administration [17]. In another study in rat, 12.0% and 9.4% of the single oral administration of radiolabeled apigenin was recovered in feces and in the intestine, respectively, within 10 days [18]. These results demonstrate that a substantial amount of apigenin passes through the small intestine and is available to interact with the gut microbiota [17,18].

The human colon gut microbiota has been found to harbor enzymes that could degrade apigenin [19,20]. In germ-free mice that consumed apigenin, apigenin and its conjugates were found to be in urine and feces, while in human microbiota-associated mice, additional degradation products and their conjugates were detected [20]. Bacterial cell extracts from *Eubacterium ramulus* and *Bacteroides distasonis* were found to be capable of converting apigenin-7-glucoside to apigenin, however, this was not the case with *Escherichia coli* (Migula) [20]. Another anaerobic bacterium isolated from human feces, *Clostridium orbiscindens*, was found to degrade apigenin to 3-(4-hydroxyphenyl)propionic acid with phloretin and naringenin as intermediates [19]. Therefore, it seems that the ability of bacteria to modify apigenin is strain specific, and not universal. In the context of the gut microbiota, degradation of apigenin and its glycosides most likely involves multiple bacteria, with complementary and overlapping functionalities.

Previous reports have found that apigenin can also affect the growth of some bacteria, inhibiting the growth of species such as *Klebsiella pneumoniae*, *Enterobacter cloacae*, *Pseudomonas aeruginosa*, *Escherichia coli (Migula)*, *Salmonella typhimurium*, *Vibrio harveyi*, *Proteus mirabilis*, and *Enterobacter aerogens*, but not, or only mildly inhibiting, *Staphylococcus aureus*, *Escherichia coli (MG 1655)*, *Bacillus subtilis* or *Proteus vulgaris* [7,8,21,22]. Although the methods employed in determining the inhibitory effects are different, nearly all studies looking at the effect of apigenin on bacterial growth have focused on pathogenic bacteria that are cultured under aerobic conditions. While these results prove that apigenin can functionally act on the growth of some bacteria, the commensal strains of the gut microbiota were not tested.

The objective of this paper was to study how apigenin, a dietary flavone with various pharmacological activities, impacts the growth and metabolism of the gut microbiota. Initially, a reductionist model was adopted to determine if apigenin would have an effect on the growth of representative gut microbial strains *Bacteroides galacturonicus*, *Bifidobacterium catenulatum*, *Lactobacillus rhamnosus* GG, and *Enterococcus caccae* by analyzing their anaerobic growth profiles for 24 h. Results of this initial study indicated that apigenin had a varying effect on the growth of the different types of bacteria. Based on these results, whether or not apigenin would have an effect on a gut microbiota community structure, or short chain fatty acid production, was examined by culturing a fecal inoculum in the presence of apigenin under in vitro anaerobic growth conditions simulating the human ascending colon region. These results indicated that apigenin may have a unique interaction with *Enterococcus*. In order to better understand this interaction at the molecular level, single molecule RNA sequencing was performed on *Enterococcus caccae*, with and without apigenin, to produce a full transcriptome profile for gene expression. Comparison of these profiles revealed the effect of apigenin on *Enterococcus caccae* at genotypic level. Taken together, these results provide a detailed analysis on the effect of apigenin on the gut microbiota community, and in particular *Enterococcus caccae*.

## 2. Results

### 2.1. The Effect of Apigenin on Single Strain Bacterial Growth

**Figure 2:**
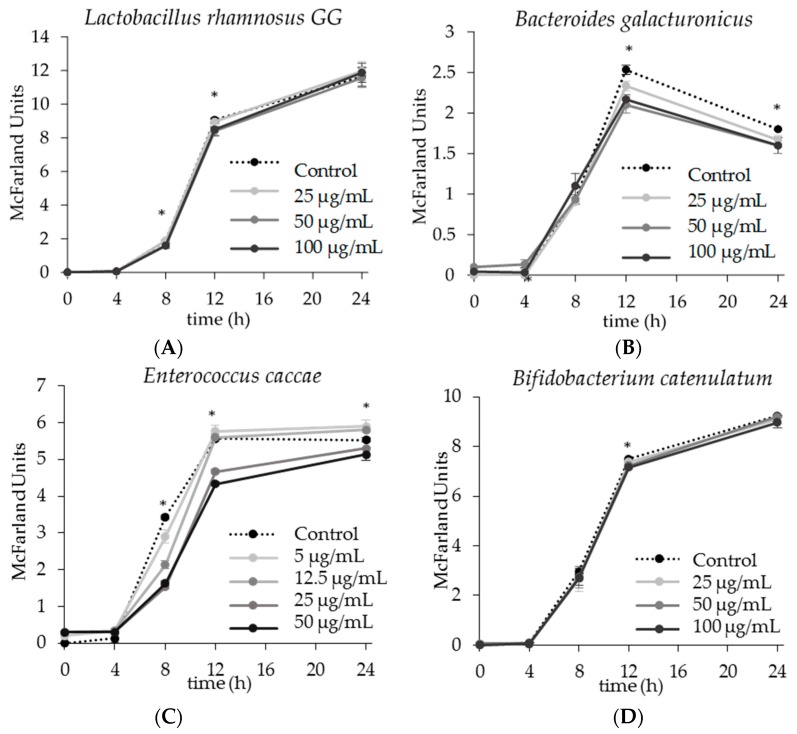
The effect of apigenin on the growth of single gut bacterial strains. Bacteria were inoculated in strain-specific media containing apigenin. McFarland unit values were determined using a densitometer at 0, 4, 8, 12, and 24 h post inoculation. The dotted line represents the control group in which bacteria grew with no apigenin added. The * mark indicates at least one experimental group was statistically significant from the control at that time point (*p* < 0.05). The 24 h growth curve with increasing concentrations of apigenin for (**A**) *Lactobacillus rhamnosus* GG; (**B**) *Bacteroides galacturonicus*; (**C**) *Enterococcus caccae*; (**D**) *Bifidobacterium catenulatum*.

The effect of apigenin on the growth of *Lactobacillus rhamnosus* GG (*L. rhamnosus* GG), *Bacteroides galacturonicus* (*B. galacturonicus*), *Enterococcus caccae* (*E. caccae*), and *Bifidobacterium catenulatum* (*B. catenulatum*) was examined by comparing the growth profile of bacteria treated with increasing concentrations of apigenin to the control group containing no apigenin (Figure 2). Such comparison revealed differences in growth over time, through lag, log, and stationary phases. Single strains of commensal gut bacteria were selected and cultured independently because this allowed comparison between different strains, and the effect of apigenin would not be masked by complex background activities that would be present in a community.

Apigenin had a minimal effect on growth of *L. rhamnosus GG.* The lowest dose of 25 µg/mL had no effect on growth at any time point (Figure 2A). The only significant change in growth was observed as a slight inhibition by 50 µg/mL of apigenin at 8 and 12 h post inoculation (Figure 2A). The McFarland units (MU) for the control at 8 and 12 h were 1.867 ± 0.058 and 9.067 ± 0.021 respectively, whereas the MU for the apigenin treated group (50 µg/mL) were 1.600 ± 0.058 and 8.400 ± 0.289 respectively. The MU readings at 8 and 12 h with 100 µg/mL of apigenin had larger variability, thus was not statistically significant (*p* > 0.05).

Growth of *B. galacturonicus* was inhibited in a dose dependent manner by the presence of apigenin at both 12 and 24 h post inoculation (Figure 2B). At 12 h, the MU readings for *B. galacturonicus* treated with apigenin were 2.333 ± 0.058, 2.100 ± 0.100, 2.167 ± 0.058 for 25 µg/mL, 50 µg/mL, and 100 µg/mL apigenin respectively, compared to the control which had an MU reading of 2.533 ± 0.058. At 24 h post inoculation these readings had decreased, with MU levels of 1.667 ± 0.058, 1.600 ± 0.026, 1.600 ± 0.026 for 25 µg/mL, 50 µg/mL, and 100 µg/mL apigenin respectively, compared to the control which had an MU reading of 1.800 ± 0.000.

The addition of apigenin had an interesting, and somewhat dichotomous effect on growth of *E. caccae* (Figure 2C). Since both 25 and 50 µg/mL of apigenin inhibited growth of *E. caccae*, a concentration of 5 and 12.5 µg/mL was also tested. At 8 h post inoculation, there was a significant, and dose dependent inhibition of growth in which the control group had an MU of 3.433 ± 0.057 and the treatment groups had MUs of 2.900 ± 0.173, 2.133 ± 0.115, 1.533 ± 0.058, 1.633 ± 0.058 for concentrations of 5 µg/mL, 12.5 µg/mL, 25 µg/mL, and 50 µg/mL apigenin respectively (Figure 2C). In fact, 25 µg/mL of apigenin was able to inhibit the growth of *E. caccae* to 52.9% of control at 8 h and 83.8% at 12 h. Interestingly, at 12 h post inoculation, only the 25 and 50 µg/mL doses inhibited growth, while the 5 and 12.5 µg/mL doses had either no effect, or a slight enhancement of growth. At 24 h post inoculation, the 25 and 50 µg/mL doses still inhibited growth, while the 5 and 12.5 µg/mL slightly enhanced the growth.

Growth of *B. catenulatum* was only minimally affected by the presence of apigenin. This can be observed in the growth curves, which illustrate no real changes in growth regardless of the dose of apigenin (Figure 2D), yet it should be noted that at 12 h post inoculation there was a statistically significant inhibition of growth by 50 µg/mL and 100 µg/mL of apigenin (Figure 2D). However, this difference was nominal; the control at 12 h had an MU of 7.500 ± 0.000, and the 50 µg/mL and 25 µg/mL treated groups had MUs of 7.233 ± 0.115 and 7.167 ± 0.100 respectively.

### 2.2. The Effect of Apigenin on a Human Gut Microbial Community In Vitro

The effect of apigenin on a human gut bacteria community was determined using batch culture fermentation. For this experiment, two bioreactors containing basal media were inoculated with the same human fecal material and run in parallel, with one bioreactor serving as a control (no apigenin) and the other as apigenin treated (100 µg/mL). Samples were harvested at 4, 8, 12, 24 and 48 h post inoculation to measure culture density, microbial composition, and SCFAs production.

**Figure 3:**
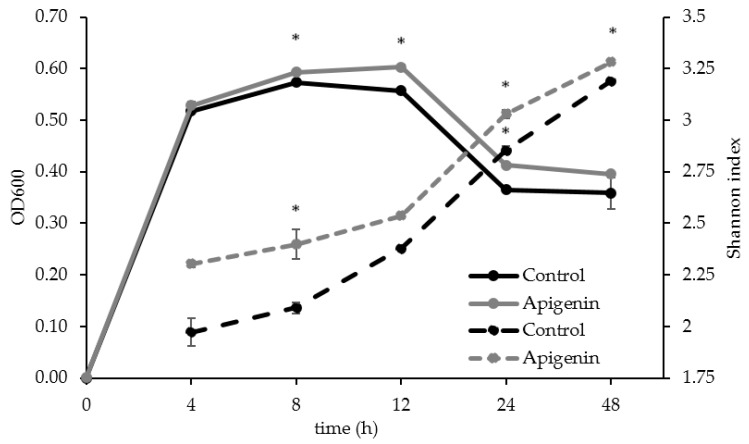
Culture density and alpha diversity over time. The culture density for the control and apigenin (100 µg/mL) treated groups based on the OD_600_ reading over time is represented using the left axis. The alpha diversity based on the Shannon index is represented using the right axis. The * mark indicates at least one experimental group was statistically significant from the control at that time point (*p* < 0.05).

Readings of culture density found that apigenin promoted overall growth of the microbial community in vitro, with a significant enhancement of growth observed for 8, 12, and 24 h post inoculation (Figure 3). The OD_600_ for the control group at 8, 12, 24, and 48 h were 0.573 ± 0.003, 0.557 ± 0.002, 0.365 ± 0.002, 0.358 ± 0.031 respectively; and for the apigenin-treated group were 0.593 ± 0.002, 0.603 ± 0.004, 0.412 ± 0.002, and 0.395 ± 0.001. Analysis of the alpha diversity for both communities using the Shannon index found that microbial diversity increased with fermentation time, with the apigenin treated group having a significantly higher Shannon index at 8, 24, and 48 h post inoculation (Figure 3).

**Figure 4:**
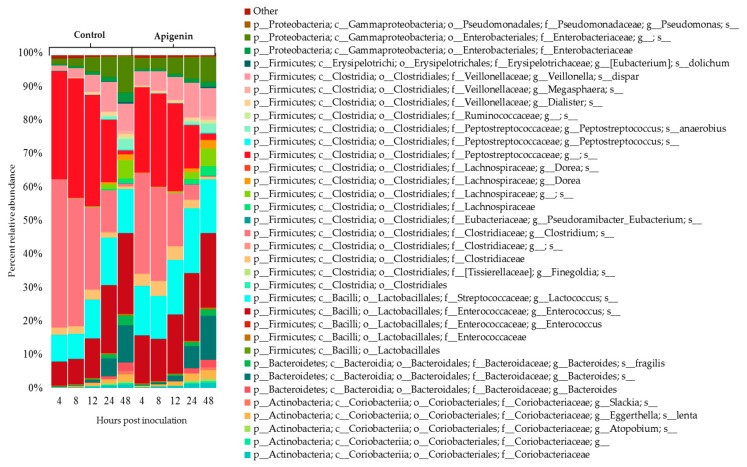
Gut bacterial community composition over 48 h. Community composition was determined based on relative abundance. OTUs that were more than 0.1% in at least one time point are presented. Diversity of the community increased over time for both the control and apigenin (100 µg/mL) treated cultures.

The community composition over time was determined in terms of relative abundance based on 16S rRNA sequencing, and the community profiles consisting of Operational Taxonomic Units (OTUs) that were more than 0.1% of the community for at least one time point were generated (Figure 4). In both the control and apigenin treated groups, there were fewer dominant OTUs at 4 and 8 h post inoculation. At these time points *Clostridium* species and Peptostreptococcaceae family were the most abundant taxa present, totaling 76.7% and 55.6% for control and apigenin groups respectively. By 24 and 48 h post inoculation the microbiota culture composition resembled that of a more of a mature community. At 48 h, 20 and 16 more OTUs grew to more than 0.1% compared to 4 h post inoculation for control and apigenin treated group, and the initially dominant *Clostridium* species and Peptostreptococcaceae family were reduced to 1.9% and 2.1% respectively. At 48 h, genera *Enterococcus*, *Lactococcus*, and *Bacteroides* were the most abundant OTUs for both the control and apigenin groups, contributing to 53.6% of the control community and 55.6% the apigenin-treated community.

**Figure 5:**
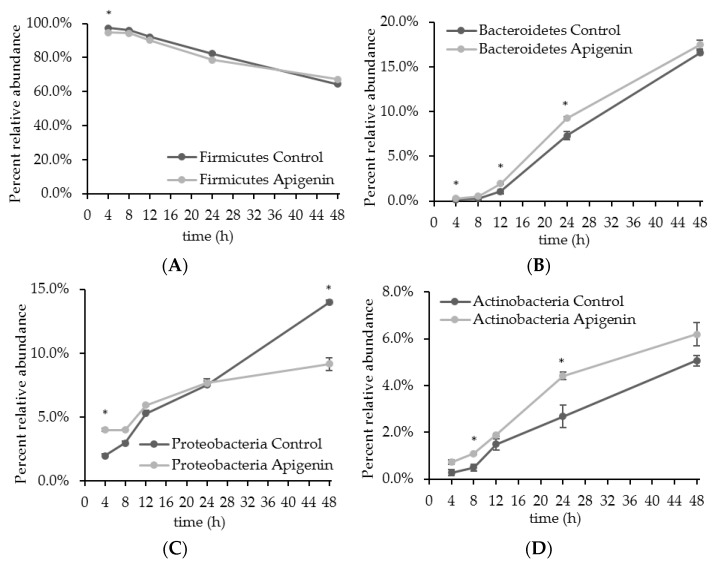
Phylum composition of the gut microbiota community over time. Bioreactors containing basal media were inoculated with human fecal homogenate, and samples harvested at 4, 8, 12, 24, 48 h post inoculation. Microbial composition of each sample was determined by 16S rRNA sequencing, and percentages of the four major phyla for both the control and apigenin-treated groups were compared at each time point. The * mark indicates the experimental group was statistically significant from the control at that time point (*p* < 0.05). (**A**) Firmicutes; (**B**) Bacteroidetes; (**C**) Proteobacteria; (**D**) Actinobacteria.

The addition of apigenin differentially affected the four major phyla in the human gut microbiota, which are Firmicutes, Bacteroidetes, Proteobacteria, and Actinobacteria (Figure 5). For both the control and apigenin-treated groups, the percentage of Firmicutes decreased with time, but remained the most dominant phylum over the course of 48 h (Figure 5A). At 4 h, Firmicutes consisted of 97.4 ± 0.1% of the control community and 94.7 ± 0.3% in the apigenin-treated community. At this early time point, apigenin significantly reduced the percentage of Firmicutes, but had no effect at any other time points.

For Bacteroidetes, the percentages in both groups increased with time (Figure 5B). At 4 h and 12 h post inoculation, apigenin significantly increased the percentage of Bacteroidetes, from 0.14 ± 0.01% and 1.07 ± 0.12% in the control to 0.28 ± 0.03% and 1.95 ± 0.10% in apigenin-treated groups (Figure 5B). As a result of the decrease in Firmicutes percentage and the increase in Bacteroidetes percentage, the Firmicutes/Bacteroidetes (F/B) ratio was decreased with apigenin treatment. At 4 h, the F/B ratio was 695.4 in the control and 338.5 in the apigenin-treated group; at 12 h the ratios were 86.0 and 46.2, respectively.

The percent of phylum Proteobacteria increased over time for both the control and apigenin treated groups (Figure 5C). The addition of apigenin resulted in an enhancement of Proteobacteria at 4 h post inoculation, with the control having 1.99 ± 0.05% Proteobacteria and the apigenin-treatment group having 4.00 ± 0.10% Proteobacteria (Figure 5C). At 8, 12, and 24 h post inoculation the amount of Proteobacteria was similar for both groups. Conversely, at 48 h, Proteobacteria percentage in apigenin-treated group, 9.14 ± 1.09%, was significantly lower than the control, 14.0 ± 0.9% (Figure 5C).

For both the control and apigenin groups, the percentage of phylum Actinobacteria increased over time (Figure 5D). At 8 h and 24 h, Actinobacteria percentages were significantly higher in the apigenin treated group (Figure 5D). The control group had 0.50 ± 0.14% and 2.67 ± 0.49% of Actinobacteria for the two time points respectively, while apigenin-treated group had 1.09 ± 0.04% and 4.41 ± 0.16%. While the difference between the groups at 48 h post inoculation is not statistically significant, there is a definite enhancement in growth for the apigenin treated group, 5.07 ± 0.23% in control group and 6.19 ± 0.49% in apigenin-treated group.

**Figure 6:**
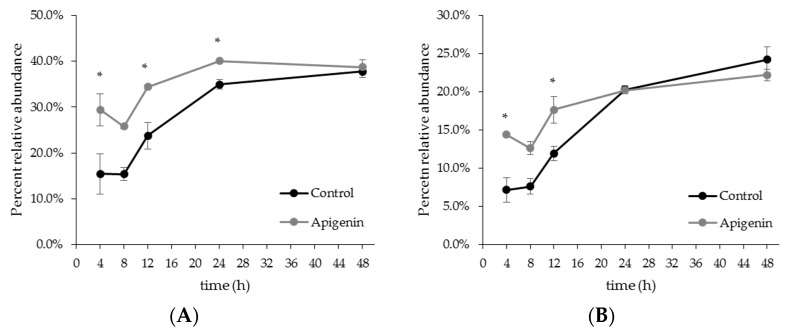
Apigenin enhances growth of order Lactobacillus and genus *Enterococcus* in a community setting. The percent relative abundance for order Lactobacillus and genus *Enterococcus* was determined based on 16S rRNA sequencing. The * mark indicates that the experimental group (100 µg/mL apigenin) was statistically significant from the control at that time point, according to a 2-tailed, Student’s *t*-test (*p* < 0.05). (**A**) Lactobacillales order; (**B**) *Enterococcus* genus.

Results from testing the effect of apigenin on *E. caccae* under axenic conditions demonstrated that the addition of apigenin influenced strain growth (Figure 2C). Therefore, whether or not this occurred in the community setting was evaluated. Since 16S rRNA sequencing of the V1V2 region is unable to distinguish between species of *Enterococcus*, the effect of apigenin on the Lactobacillales order and *Enterococcus* genus was examined (Figure 6). Analysis of the growth trend revealed that proportions of order Lactobacillales were significantly enhanced at 8, 12, and 24 h post inoculation for the apigenin treated group (Figure 6A). Looking at the genus level, the addition of apigenin resulted in a significant increase for *Enterococcus* at 4 and 8 h post inoculation (Figure 6B). At 4 h, the control proportion was 7.18 ± 1.61% for *Enterococcus* genus, and the apigenin-treated proportion was 14.4 ± 0.05%. At 8 h, the proportions were 7.61 ± 1.04% for the control; 12.6 ± 0.8% for the apigenin-treated group.

The growth trends were almost identical between the Lactobacillales order and *Enterococcus* genus (Figure 6). This is because the Lactobacillales order was largely composed of *Enterococcus* in this experiment. Interestingly, the effect of apigenin was different from when *E. caccae* was cultured alone. While the single strain growth was inhibited by apigenin effectively, apigenin supported *Enterococcus* genus growth to a higher percentage in a community setting.

### 2.3. Apigenin Influences Short Chain Fatty Acids Production in a Community Setting

**Figure 7:**
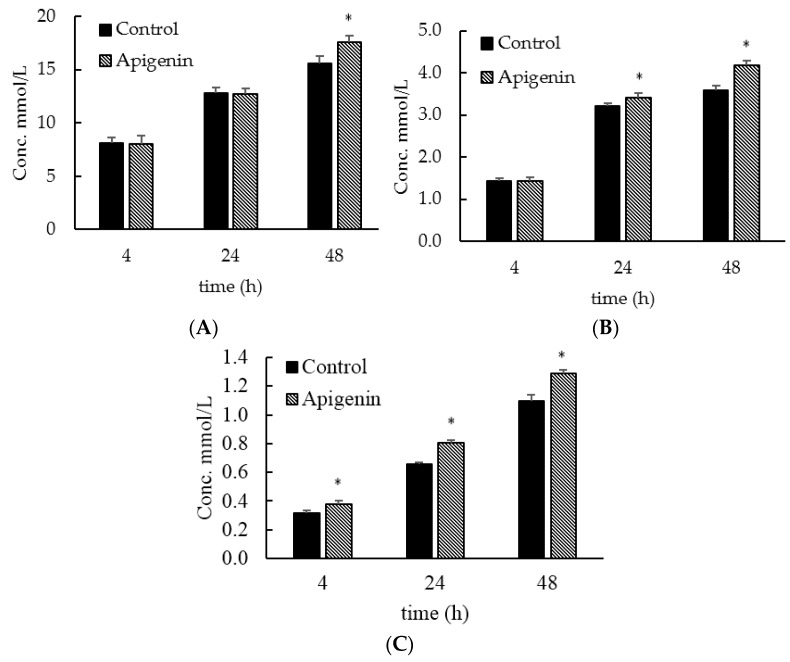
Major short chain fatty acid production by the gut microbial community over time. mmol/L is millimolar per liter. Amounts of the short chain fatty acids acetate, propionate, and butyrate were measured using a GC/MS. The * mark indicates the experimental group was statistically significant from the control at that time point (*p* < 0.05). (**A**) Acetate; (**B**) Propionate; (**C**) Butyrate.

The most abundant short chain fatty acids (SCFAs) detected in the culture were acetate, propionate, and butyrate. Other SCFAs, including 2-methylpropionate, 2-methylbutyrate, pentanoic acid, 2-methylpentanoic acid and 4-methylpentanoic acid were also detected, but only in low or trace concentrations. The amounts of acetate, propionate, and butyrate produced all increased with time for both the control and apigenin-treated groups (Figure 7). Acetate production was significantly higher in the samples containing apigenin at 48 h post inoculation, 17.546 ± 0.597 mmol/L, compared to the control, 15.539 ± 0.772 mmol/L (Figure 7A). Propionate concentration was higher in the apigenin-treated samples at 24 and 48 h post inoculation (Figure 7B). The control group concentrations were 3.221 ± 0.055 mmol/L, 3.579 ± 0.120 mmol/L, and apigenin-treated concentrations were 3.413 ± 0.108 mmol/L and 4.171 ± 0.121 mmol/L for 24 and 48 h, respectively. Apigenin-treated gut microbiota produced more butyrate for all three time points tested, with the control, in the order of 4, 24, and 48 h, producing 0.318 ± 0.015 mmol/L, 0.658 ± 0.014 mmol/L, and 1.097 ± 0.045 mmol/L and apigenin-treated group producing 0.381 ± 0.021 mmol/L, 0.803 ± 0.022 mmol/L, and 1.287 ± 0.027 mmol/L of butyrate (Figure 7C).

### 2.4. Apigenin-Induced Changes to the Genetic Expression Profile of Enterococcus caccae

The gene expression profiles of *E. caccae* grown with and without the presence of 25 µg/mL apigenin were assembled and compared. A total of 764 individual genes were identified; 141 of these had expression levels significantly different from the control (*p* < 0.05). Among the 141 genes, 97 were up-regulated, and 44 were down-regulated. For brevity, only genes with a greater than 1.5-fold change are considered in the Discussion. Setting the threshold at a 1.5-fold change, 60 genes were up-regulated and 30 were down-regulated. Their fold change, number, and brief description of function can be found in Table 1. Of the up-regulated genes, 17 were hypothetical proteins, and 18 of the down-regulated genes were hypothetical proteins.

## 3. Discussion

Apigenin is a flavonoid widely consumed in the diet, yet little is known regarding its ability to alter the gut microbiota. Using a reductionist model, the effect of apigenin on the growth of single gut commensal bacteria was first analyzed (Figure 2). These results demonstrated that apigenin is able to influence growth of some gut microbes, but that this effect is species dependent. Clearly, growth of *L. rhamnosus GG* and *B.catenulatum* were not affected by apigenin. This is not surprising, considering that *L. rhamnosus GG* is specifically used as a probiotic because it is unaffected by harsh conditions such as acid and bile [23] and *B. catenulatum* is unaffected by other polyphenols, such as quercetin [24]. It is possible that *L. rhamnosus GG* and *B. catenulatum* may respond to apigenin at a higher concentration, however, the low solubility of apigenin in media limits the concentration to no more than 100 µg/mL.

Apigenin effectively inhibited growth of both *E. caccae* and *B. galacturonicus.* Among the four strains, *B. galacturonicus* is the only Gram-negative strain. However, the observed inhibitory effect is not considered to be determined by Gram stain status. This was concluded based on the observation that both Gram-positive and Gram-negative strains were inhibited to some extent (*E. caccae* and *B. galacturonicus*, respectively), however, not all G+ strains were inhibited.

The effect of apigenin on *E. caccae* specifically revealed a divergent pattern between the treatment groups. For the 5 and 12.5 µg/mL apigenin groups, inhibition was observed during the early exponential phase. Yet, the rate of cell density increase was similar between the control and apigenin treated groups after 8 h, with a potential enhancement of growth due to the addition of apigenin (Figure 2C). However, for the 25 and 50 µg/mL apigenin groups, growth was significantly inhibited after 4 h post inoculation (Figure 2C). A possible reason for this difference is that the lower concentrations of apigenin were able to inhibit *E. caccae* growth prior to 12 h just enough so that there were more nutrients left in the media, and less metabolic waste, allowing for growth to surpass the control group at 12 and 24 h post inoculation. These results indicated that *E. caccae* is able to either degrade apigenin into byproducts that are no longer inhibitory, or overcome this inhibition through genetic regulation.

Species of the *Enterococcus* genus have been identified in the human and animal gastrointestinal (GI) tracts, as well as in traditional fermented food and dairy products, and in various extraenteric environments [25,26]. *Enterococcus* is considered a commensal opportunist and they emerged in the 1970s as some of the leading causes of multidrug-resistant, hospital-acquired infections [25]. Considering that *Enterococci* have been known to cause multidrug-resistant infections, and how close *E. caccae* is related to the most abundant *Enterococcus* species in the human gut, the inhibitory effect of apigenin provides an opportunity for developing new antibiotics.

The results of the single strain testing revealed that the ability of apigenin to influence growth of the gut commensal bacteria was individualistic. Some species were affected and others were not, and among those affected the growth profiles were modified in different ways. Therefore, whether or not apigenin would have an effect on a complete gut community was questioned. Since a community has hundreds of species that are able to interact, a reductionist model cannot be used to determine a community effect. To examine the effect of apigenin on a complete community, bioreactors were set to simulate the ascending colon region only using a batch culture model. The ascending colon region was chosen because it is where the apigenin in foods first interacts with the gut bacteria; the other colon regions would be interacting with a lower concentration of apigenin or its degradation products as it has been passed from previous regions. It should be noted that the effects of apigenin could very possibly vary across different regions or microenvironments of the colon.

In batch culture of the microbiota, it was observed that in both the control and apigenin groups, microbial diversity increased with time, represented by an increasing number of OTUs of higher abundance over the course of 48 h. This is not surprising since after inoculation the bacteria were able to enter into an exponential growth phase, allowing the community to mature and species that were below detection level in the fecal sample to develop. The amount of growth for both the control and apigenin was similar, with enhancement by apigenin, as determined by optical density and alpha diversity using the Shannon index. These observations are important because they indicate that apigenin did not inhibit growth and maturation of the gut microbiota, but may function to enhance overall diversity of the community.

There were some statistically different changes between the control and apigenin treated groups at the phylum level. However, other than for phylum Proteobacteria at 48 h, those differences were not as drastic as the inhibitory effect observed when *E caccae* was tested under axenic conditions, a reduction of 47.1% compared to control with presence of 25 µg/mL of apigenin. This is not entirely surprising since phyla contain a large number of OTUs, which make them more robust than a single strain, and the effect of apigenin may occur at an intra-phylum level. However, the small changes noted did result in a change to the Firmicutes/Bacteroidetes (F/B) ratio, which was lower in the apigenin-treated microbiota. Since a higher F/B ratio has been associated with obesity [27], lowering the F/B ratio indicates that apigenin has the potential to shift the microbiota away from the ratio associated with obesity.

Interestingly, apigenin did not inhibit growth of *Enterococci* in the community. On the contrary, growth of genus *Enterococci* was enhanced with apigenin at 4 and 8 h post inoculation. This is opposite to what happened when apigenin was tested with *E. caccae* alone. One possible explanation is that apigenin was degraded or taken up by other species in the community, and as a result *Enterococci* were exposed to less apigenin. Other bacteria more susceptible to apigenin were inhibited and thus allowed for an enhancement of *Enterococci*. It is also possible that the *Enterococci* in this fecal sample were not sensitive to apigenin. Determination if this is the case would require identification of the *Enterococcus* species and examination of their growth in the presence of apigenin.

The gut microbiota community not only resides in the colon, but is a functional part of the environment. Fermentation of substrates by the gut microbiota produces short chain fatty acids, which are absorbed by the colonic cells. Therefore, the effect of apigenin on the gut microbiota may not be limited to community structure, but could also result in changes to the production of SCFAs. In this case, it is not just about an effect on who is there, but what they are doing, and what they are producing.

The results of this study are in accordance with previous findings in that the most abundant SCFAs produced were acetate, followed by propionate, and butyrate [28]. In this study, the production of all three major SCFAs were enhanced by apigenin. SCFAs participate in the regulation of mucin secretion. Goblet-cell-specific Mucin 2 (MUC-2) is the most prominent mucin secreted by intestinal epithelial cells. It has been demonstrated that butyrate and propionate both induce an increase in MUC-2 mRNA levels [29]. It is known that prostaglandins (PG) enhance mucin secretion and are key players in mucoprotection. SCFA can differentially regulate PG production, promoting the production of more potent Prostaglandin E1 (PGE1) over PGE2, thus stimulating MUC-2 expression in intestinal epithelial cells [30]. Apigenin, by enhancing the production of butyrate and propionate, may stimulate mucin production. This would be protective for the gut lining and facilitate mucin-dependent gut bacteria growth, e.g., *Akkermansia muciniphila*. However, this effect is yet to be tested in vivo.

Butyrate, which was significantly higher in apigenin-treated samples at all three time points, is almost completely consumed by the colonic epithelium, and it is a major source of energy for colonocytes [31]. It has been estimated that 70% to 90% of butyrate is metabolized by the colonocytes [32], where butyrate promotes cell differentiation, cell-cycle arrest and apoptosis of transformed colonocytes. Sodium butyrate exerts an anti-proliferative activity on many cells types, showing preventive effects on colon cancer and adenoma development [32]. Butyrate also stimulates immunogenicity of cancer cells [32]. Therefore, it is logical to propose that a diet containing apigenin may contribute to health by increasing the amount of butyrate produced by the microbiota.

The results of single strain and community studies demonstrated that apigenin had a divergent effect on growth of *E. caccae*, inhibiting growth in a single strain setting and enhancing growth in a community setting. In order to elucidate how apigenin and *E. caccae* may interact at the genetic level, resulting in a change in growth status, RNA expression was evaluated. Among all the 764 genes identified, 290 were described as hypothetical proteins, including the most upregulated and the most downregulated ones, thus their identities and functions are unknown. Setting the threshold at a fold change of 1.5 times among those are not hypothetical proteins, 43 genes were up-regulated and 12 were down regulated.

It has been previously indicated that apigenin affects nucleic acids, type II fatty acids and d-alanine:d-alanine ligase in bacteria. Related genes could be upregulated by apigenin as the bacteria compensates for the inhibition exerted on the end products, e.g., enzymes. The results of the gene expression for this experiment demonstrated that Tyrosine recombinase XerC, which is responsible for chromosome dimer resolution during DNA replication [33], was upregulated by 2.8 fold; excinuclease ABC subunit B, NrdR family transcriptional regulator, and UvrABC system protein A, which are involved in DNA repair were upregulated by 2, 1.7, and 1.7 fold respectively [34,35,36,37]. This supports the findings that nucleic acids are damaged by apigenin. Results also showed that genes for an acyl carrier protein and 3-oxoacyl-ACP synthase III, two components involved in Type II fatty acid synthesis [38,39,40,41], were upregulated by 2.6 and 2.3 fold, respectively, which indicates that apigenin alters the type II fatty acid biosynthesis. d-Alanine-poly(phosphoribitol) ligase subunit 2, which participates in cell wall and cell membrane metabolism [42] was also upregulated by 1.6 fold, which could be a result of the inhibitory effect of apigenin targeting d-alanine ligase.

The results of gene expression analysis in this study indicate that cell membrane and cell wall synthesis is likely a major target of apigenin in *Enterococcus caccae*. Both the d-alanine:d-alanine ligase and Type II fatty acid synthetic pathway are involved in cell membrane/wall synthesis. The Type II fatty acid synthetic pathway is the principal route for membrane phospholipid acyl chains production [43]. Several other genes related to cell membrane or cell wall metabolism were also found to be upregulated, including the following: glycerophosphoryl diester phosphodiesterase, upregulated 2.9-fold, is involved in phospholipid metabolism [44,45]; phosphoglyceroltransferase, upregulated 2.1-fold, is involved in lipoteichoic acid biosynthesis [46,47]; acyltransferase, upregulated 2.1-fold, is involved in cell wall peptidoglycan synthesis [48,49]; penicillin-binding protein and penicillin-binding protein 2B, upregulated 1.5- and 1.6-folds respectively, are involved in cell wall synthesis [50,51]; *N*-acetylmuramoyl-l-alanine amidase, upregulated 1.7-fold, participates in cell separation [52]; undecaprenyl pyrophosphate synthase, upregulated 1.8-fold, participates in peptidoglycan biosynthesis [53].

Several general stress response genes were also found to be upregulated due to apigenin treatment. The MarR family transcriptional regulator was upregulated 2.5-fold compared to the control [54], spx/MgsR family transcriptional regulator was upregulated by 2.1-fold [55,56], and the cold-shock protein was upregulated by 1.7-fold. This is a sign that the cell was turning on responsive protection mechanisms to overcome damage caused by apigenin. As a result, fewer energy and resources were used for cell growth and division.

Protein chaperones facilitate correct folding of proteins and help with the refolding of misfolded proteins [57]. Several protein chaperones and elements involved in controlling protein quality were upregulated: chaperonin by 2.2-fold; molecular chaperone GroEL by 1.7-fold, chaperone protein ClpB by 1.6-fold, and ATP-dependent protease ATPase subunit HslU by 2.3-fold, and ATP-dependent protease subunit HslV by 2.2-fold. The latter two are involved in protein quality control [58]. This could indicate an increase in misfolding of proteins in the cell as a result of apigenin treatment. One possibility is that the cell wall and cell membrane protein are being produced, but misfolded. This would explain the increase in transcription of cell wall and cell membrane genes, without the production of the protein themselves. 

Among the genes that were down-regulated, two participate in iron-sulfur (FeS) cluster formation and one is an element in FeSr biosynthesis: FeS assembly protein SufB by 1.6-fold; iron-sulfur cluster assembly scaffold protein by 1.6-fold; and SufS family cysteine desulfurase by 1.6-fold. Fe-S cluster is regulated under homeostatic control, meaning that it is regulated according to cellular requirements [59]. A decrease in expression of related genes could be the result, or the cause, of slower growth rate in apigenin-treated cells, as iron is an important metal ion for bacterial growth.

The results of this study demonstrated that apigenin was able to influence growth of some gut microbes in a species dependent manner: effectively inhibiting growth of both *E. caccae* and *B. galacturonicus*, but not *L. rhamnosus GG* or *B.catenulatum.* In apigenin-treated microbiota, phylum percentages were different from the control, with a lowered Firmicutes/Bacteroidetes (F/B) ratio. Contrary to the inhibition on *E. caccae* when cultured alone, apigenin enhanced growth of *Enterococci* in the community. Production of short chain fatty acids was promoted by apigenin. Gene expression profiles changed with apigenin-treatment showing that apigenin had a negative effect on cell wall/cell membrane synthesis, protein folding, and triggered overall stress response.

## 4. Materials and Methods

### 4.1. Media Preparation

Difco^TM^ Lactobacilli MRS broth (Becton, Dickinson and Company, (Franklin Lakes, NJ, USA) was made by resuspending 55.00 g of MRS powder in a total volume of 1 L deionized, distilled water. The solution was heated with agitation until the powder was completely dissolved.

*Bacteroides galacturonicus* medium was made using the following ingredients: Sodium polygalacturonate 4.00 g, trypticase peptone 5.00 g (Becton Dickinson), yeast extract 2.50 g, MgSO _4_ × 7 H_2_O 2.50 g, CaCl _2_ × 2 H_2_O 0.15 g, FeSO_4_ × 7 H_2_O 20.00 mg, (NH_4_)_2_SO_4_ 1.40 g, l-cysteine 1.00 g, resazurine stock solution 1.5 mL, NaHCO_3_ 40 mL 5% (*w*/*v*) solution, in a final volume of 1 L deionized, distilled water. Final pH was adjusted to 7.1 using 10 M NaOH or 37% HCl.

Tryticase Soy Yeast Extract Medium for *Enterococcus caccae* was made with the following ingredients: 30.00 g trypticase soy broth (Becton Dickinson), and 3.00 g yeast extract, in a final volume of 1 L deionized, distilled water. Final pH was adjusted to 7.0–7.2 using 10 M NaOH or 37% HCl.

*Bifidobacterium catenulatum* medium was made with the following ingredients: Casein peptone tryptic digest 10.00 g (Becton Dickinson), yeast extract 5.00 g, meat extract 5.00 g, Bacto Soytone 5.00 g (Becton Dickinson), glucose 10.00 g, K_2_HPO_4_ 2.00 g, MgSO_4_ × 7 H_2_0 0.20 g, Tween 80 1.00 mL, NaCl 5.00 g, cysteine-HCl × H_2_O 0.50 g, resazurin stock solution 1 mL, salt solution 40 mL in a final volume of 1 L deionized, distilled water. Cysteine-HCl × H_2_O was added after the medium had been boiled and cooled. Final pH was adjusted to 6.8 using 10 M NaOH or 37% HCl. Resazurin stock solution was made by dissolving 0.1 g resazurin in 100 mL deionized, distilled H_2_O. Salt solution was made by dissolving the following in 1 L deionized, distilled water: CaCl_2_ × 2 H_2_O 0.25 g, MgSO_4_ × 7 H_2_O 0.50 g, K_2_HPO_4_ 1.00 g, KH_2_PO_4_ 1.00 g, NaHCO_3_ 10.00 g, NaCl 2.00 g, then filter sterilized.

Basal medium [60] was made fresh before use with the following ingredients: Peptone 2 g (Becton Dickinson), yeast extract 2 g, l-cysteine 0.5 g, NaCl 0.1 g, K_2_HPO_4_ 40 mg, KH_2_PO_4_ 40 mg, MgSO_4_ × 7H_2_O 10 mg, CaCl_2_ × 2H_2_O 6.7 mg, Tween80 2 mL, resazurine stock solution 1.5 mL (resazurine stock solution: resazurine 0.1 g, deionized, distilled water 100 mL), with a final volume of 1 L deionized, distilled water. The final pH was adjusted to 5.8 using 10M NaOH or 37% HCl.

All broths were autoclaved at 121 °C for 30 min. Before being transferred into a Bactron anaerobic chamber to cool overnight, the broth was boiled under negative pressure using nitrogen gas for 10 min to remove any oxygen from the solution. All above anaerobic broths were made fresh every two to three weeks and stored at room temperature under oxygen free conditions after cooling in the anaerobic chamber. All ingredients were purchased from Sigma unless otherwise labeled.

### 4.2. Apigenin Stock Solutions

Apigenin (≥98%, Item No. 10010275) was purchased from Cayman Chemical Company (Ann Arbor, MI, USA) and was stored at −20 °C prior to use. For the single strain bacteria growth tests, stock solutions were made at 500 times the final concentration in an Eppendorf tube with DMSO (Sigma, St. Louis, MO, USA) before being transferred into the anaerobic chamber. Each tube was vortexed to ensure homogenization. Stock solutions were diluted with media at a 1:9 volume ratio and a final volume of 100 µL of each diluted solution was injected into a sealed Hungate tube (Chemglass, Vineland, NJ, USA) containing 5 mL of media. Final concentrations of apigenin tested were 5, 12.5, 25, 50, and 100 µg/mL.

For use in the batch culture experiment, apigenin was made into stock solutions 500 times the final concentration in DMSO to increase solubility before being transferred into the anaerobic chamber, as described above. The stock was then diluted with basal medium at 1:1 ratio in the anaerobic chamber to avoid corrosion of pure DMSO on plastics. Final concentration for apigenin in each bioreactor was 100 µg/mL.

### 4.3. Single Strain Bacteria Preparation

*Lactobacillus rhamnosus* GG (LGG, 53103)) was purchased from ATCC (Manassas, VA, USA). The other four strains were ordered from the company Deutsche SammLung von Mikroorganismen und Zellkulturen GmbH (DSMZ, Braunschweig, Germany): freeze dried ampoule of 3978, type strain; *Bacteroides galacturonicus* N6, freeze dried ampoule of 19829, type strain; *Enterococcus caccae* SS-1777 (*E. caccae*), freeze dried ampoule of 16992, type strain, *Bifidobacterium catenulatum* B669 (*B. catenulatum*). Each bacterial strain was recovered from frozen aliquots in strain specific broths (described above) and grown overnight in the anaerobic chamber at 37 °C sequentially at least twice prior to use in order to ensure recovery from freezing.

### 4.4. Growth Curve Measurement of Single Strain Bacteria

All steps of microbial culture were performed using a Bactron anaerobic chamber to ensure oxygen free conditions. For the single strain experiments, anaerobic broth was aliquoted into Hungate tubes prior to starting an experiment, 5 mL per tube. The tubes were sealed with a rubber septum (Chemglass) and screw cap lid (Chemglass), and stored at room temperature in the anaerobic chamber until needed. Each Hungate tube containing 5 mL pre-aliquoted broth was injected with 100 µL of the corresponding diluted stock solution of apigenin with a 1 mL needle and a 25-gauge syringe.

The cultures of bacteria grown overnight were diluted in their specific broth to 0.5 McFarland units above background. Each 5 mL Hungate tube containing the appropriate anaerobic broth and the desired concentration of apigenin was injected with 100 µL of this culture using a 1 mL syringe and a 25-gauge needle. Each Hungate tube was briefly vortexed after injection to ensure proper distribution and the McFarland units for each culture were determined using a DEN-1 densitometer (Grant Instruments, Cambridge, UK). After adding apigenin and the bacteria, the McFarland units were determined as the time 0 read. The cultures were then placed into the anaerobic incubator set to 37 °C. Each culture was removed temporarily from the incubator at 4, 8, 12, and 24 h post inoculation, briefly vortexed, and the McFarland units measured using a densitometer. For each strain at each concentration of apigenin, six Hungate tubes of broth were used. Three tubes were designated as a broth control, consisting of broth containing the desired concentration of apigenin only. The other three tubes were designated as the experimental group, consisting of broth containing apigenin which were also inoculated with bacteria. The sets with DMSO only, with 0 µg/mL of apigenin, were designated as the control group.

### 4.5. Figures and Statistics for Single Strain Bacteria Growth Curve

For the growth of a single strain bacteria, the McFarland readings from each group were adjusted by subtracting the broth control read from the experimental read. The adjusted numbers were plotted in a growth curve as McFarland units above broth-only over time. For each time point, a 2-tailed, unpaired homoscedastic Student’s *t*-test was run to determine if the difference between the control and the experimental groups was statistically significant, *p* < 0.05.

### 4.6. Inoculum Preparation for Batch Culture Experiment

In order to prepare a fecal inoculum to use in the batch culture experiment, 630 mL of basal medium was sterilely transferred into a bioreactor after the empty vessel had been flushed sterilely with nitrogen for 10 min. The transferred medium was then sparged with nitrogen for 20 min at 37 °C to achieve anaerobic conditions. Resazurin color indicator was used to confirm anaerobic conditions. The temperature was maintained at 37 °C with constant stirring, and the pH was computer controlled, and set to 5.8 ± 0.1 using 0.5 M HCl and 0.5 M NaOH.

After the removal of oxygen from the basal media, 70 mL of defrosted human gut microbiota preparation (OpenBiome; Somerville, MA, USA) was added into the bioreactor, mixed three times with a syringe and allowed to grow for 16 h overnight. The donor of the gut microbiota preparation was a healthy female between 25–45 years of age, with a normal BMI, who had been following a non-vegetarian American diet, and had been antibiotic free for at least six months. The preparation was stored at −80 °C and thawed at 37 °C on the day of use. After complete thawing and thorough mixing, half of the preparation was used in the first replicate of batch fermentation experiment. The other half was frozen at −80 °C immediately after aliquoting and was used in the second replicate of the batch fermentation experiment.

### 4.7. Batch Culture Fermentation

Batch culture was performed in a series of bioreactors using SHIME^®^, Simulator of the human intestinal microbial ecosystem (Ghent University-ProDigest, Ghent, Belgium). SHIME^®^ was originally designed to simulate the complete GI tract, but for this experiment it was set up to simulate only the ascending colon. The glass bioreactors have double jackets allowing heating water to circulate across all the vessels, maintaining 37 °C. The lids were assembled with two head space flush ports, one media sparging port, one sample port for removing medium during the experiment, acid and base ports, and a pH probe. The entire system was sterilized before use. pH probes were sterilized with 75% ethanol and the rest were autoclaved at 121 °C for 15 min.

Prior to the start of the experiment, 540 mL of basal medium was sterilely transferred into each bioreactor using the same method as described above. Apigenin stock solution was made as described above and added to the vessel using a syringe. One vessel received DMSO only was deemed as the control group. After adding the basal medium and the apigenin, 3 mL of sample was harvested and tested for OD_600_ in a G10S UV-Vis spectrophotometer (Thermo Fisher, Waltham, MA, USA).

At time 0 h, 60 mL of inoculum prepared overnight was added into each bioreactor. Nitrogen was sparged through each vessel for 10 min to ensure anaerobic condition. Growth condition was maintained for 48 h at 37 °C with constant stirring and pH controlled at 5.8 ± 0.1. Samples were harvested at 4, 8, 12, 24, and 48 h post inoculation and OD_600_ values were measured. Another set of samples were centrifuged at 5000× *g* for 10 min and the supernatants were then filtered with 0.20 Micron PES filters (Corning, Corning, NY, USA). Both the pellets and filtered supernatant were immediately stored at −80 °C until further analysis.

### 4.8. DNA Extraction

A DNA extraction was performed on harvested samples using a bead beater-CTAB extraction method. For each sample, 500 µL CTAB buffer and 500 µL of phenol-chloroform-isoamyl alcohol (PCI) were added to a pellet spun down from the collected samples. After vortexing, all liquid was transferred to a bead-beating tube, prefilled tube kit, with Triple-PureTM High Impact Zirconium Beads of diameter 0.1 mm (Benchmark Scientific, Sayerville, NJ, USA). Bead beating tubes were placed in a BeadBug^TM^ microtube homogenizer (Benchmark Scientific) for two, 20-s bead-beating rounds, with a 20 s interval in between. Tubes were centrifuged at 3000× *g* for 5 min. 300 µL of the supernatant were transferred to a new 1.5 mL Eppendorf tube. Five hundred µL of CTAB buffer were added to the original bead-beating tube. Tubes were homogenized again with two rounds of 20-s bead-beatings and a 20 s interval in between. Tubes were centrifuged at 3000× *g* for 5 min. 300 µL of the supernatant were transferred and combined with the previous 300 µL. Six hundred µL of Chloroform-isoamyl alcohol(CI) were added into the 1.5 mL Genemate tube, inverted and spun for 10 s with a minifuge (VWR, Radnor, PA, USA). Five hundred µL of the upper aqueous phase were transferred to a new 2 mL Eppendorf tube. One thousand µL PEG-6000 precipitation solution were added and incubated at room temperature for 2 h. Tubes were then centrifuged for 10 min at 18,200× *g*. Supernatant was removed and the pellet was washed with 1 mL ice cold 70% ethanol. Tubes were centrifuged again for 10 min at 18,200× *g*. Supernatant was removed and the tubes were air-dried for 30 min under laminar flow in a Biosafety Cabinet (Labconco, Kansas City, MO, USA). Samples were then eluded with 75 µL of PCR grade water (Roche, Brandford, CT, USA).

CTAB buffer was made by dissolving 4.20 g K_2_HPO_4_ and 4.091 g NaCl in 200 mL deionized, distilled water. The solution was autoclaved at 120 °C for 30 min. The day before use, 10 g of Hexadecyltrimethylammonium bromide (CTAB) was added to the autoclaved solution and heated to 60 °C with agitation until the particles were completely dissolved. PEG-6000 precipitation solution was made by completely dissolving 300 g of polyethylene glycol 6000 (Alfa Aesar, Haverhill, MA, USA) and 93.5 g NaCl in 1000 mL deionized, distilled water. The solution was autoclaved at 120 °C for 30 min and later stored at room temperature.

### 4.9. DNA Sequencing

Barcoded PCR primers annealing to the V1-V2 region of the 16S rRNA gene was used for library generation using the primer sequences 27F (AGAGTTTGATCCTGGCTCAG) and 338R (TGCTGCCTCCCGTAGGAGT) [61,62]. PCR reactions containing 50 nanograms of DNA and 10 pM of each primer was carried out in quadruplicate using high fidelity Accuprime Taq (Invitrogen, Carlsbad, CA, USA). The resulting 16S rDNA amplicons were purified using a 1:1 volume of Agencourt AmPure XP beads (Beckman-Coulter, Brea, CA, USA), quantified using PicoGreen, pooled in equal amounts, and sequenced on the Illumina MiSeq (San Diego, CA, USA) using 2 × 250 bp chemistry. Extraction blanks and DNA free water were subjected to the same amplification and purification procedure to allow for empirical assessment of environmental and reagent contamination. Positive controls, consisting of eight artificial 16S gene fragments synthesized in gene blocks and combined in known abundances, were also be included [63].

### 4.10. Bioinformatics Processing

Sequence data was processed using QIIME version 1.9 [63]. Read pairs were joined to form a complete V1V2 amplicon sequence if they had a minimum overlap of 35 base pairs and maximum overlap difference of 15%. Then they were quality filtered with a minimum quality threshold of Q20. Operational Taxonomic Units (OTUs) were selected by clustering reads at 97% sequence similarity [64]. Taxonomic assignments were generated by comparison to the Greengenes reference database [65], using the consensus method implemented in QIIME. A phylogenetic tree was inferred from the OTU data using FastTree [66]. For each time point, a 2-tailed, unpaired homoscedastic Student’s *t*-test was run to determine if the percentage difference of a given OTU, genus, order, or phylum between the control and the experimental groups was statistically significant, *p* < 0.05.

### 4.11. RNA Extraction

*Enterococcus caccae* cultured with DMSO only and 25 µg/mL of apigenin were harvested at 8 h post inoculation. Two samples were collected from each group, centrifuged at 5000× *g* for 5 min at 4 °C to pellet the cells, and the supernatant discarded. RNA from these samples was purified using an Ambion PureLink^®^ RNA Mini Kit (Cat no. 12183018A, ThermoFisher, Waltham, MA, USA).

### 4.12. RNA Sequencing: rRNA Removal, cDNA Synthesis, Poly A Tailing and Blocking

Ribosomal RNA depletion of the bacterial total RNA was performed using the Illumina Bacterial Ribo-Zero kit (#MRZMB126). Magnetic beads were prepared by vortexing and aliquoting 225 µL per reaction into RNase-free tubes, placing on magnetic stand, and removing supernatant. Beads were washed twice with RNase free water, followed by addition of 65 µL of Magnetic Bead Resuspension solution and 1 µL of RiboGuard RNase Inhibitor.

RNA was treated with rRNA Removal solution by combining ~1 µg of total RNA with RNase-free water, 4 µL of Ribo-zero rRNA Reaction Buffer, and 8 µL Ribo-zero Removal Solution to a total of 40 µL, and incubating at room temperature for 5 min.

The probe-hybridized RNA solution was then transferred to the magnetic beads and the solution was mixed, incubated at room temperature for 5 min, and subsequently at 50 °C for 5 min. Tubes were removed from the heat and placed on the magnetic stand. Supernatant containing 90 µL of rRNA depleted sample was removed and placed on ice until purified.

Ethanol precipitation was performed by adjusting the volume of sample to 180 µL with water, followed by addition of 18 µL of 3M sodium acetate, 2 µL of glycogen (10 mg/mL), and 600 µL of 100% ethanol. This solution was gently vortexed and placed at 80 °C for overnight. After approximately 16 h, the solution was centrifuged at 10,000× *g* for 30 min and supernatant was carefully removed. The pellet was washed twice with a 70% ethanol solution, air dried, and dissolved in RNase free water. Concentration was measured using the nanodrop.

First strand cDNA synthesis was carried out by initially incubating 7 µL of sample plus water (~100 ng RNA) at 95 °C for 5 min and chilling on ice for 2 min. While on ice, 5 µL of 50 ng/µL random hexamers and 1 µL of dNTP mix were added to the RNA and the mixture was incubated at 65 °C for 5 min. While on ice, 4 µL of 5 × buffer and 1 µL of 0.1 M DTT were mixed in to the solution and incubated at 15 °C for 20 min. Finally, 1 µL of RNase Inhibitor and 1.5 µL of Superscript III Reverse Transcriptase (Invitrogen #18080-044) were added. The following program was run on the thermal cycler: 25 °C for 10 min, 40 °C for 40 min, 55 °C for 50 min, and 85 °C for 5 min. Upon completion, 1 µL each of RNase H and RNase If was used to digest the RNA strand at 37 °C for 30 min. Performa DTR Gel filtration cartridges (#42453, EdgeBio, Gaithersburg, MA, USA) were used to purify the cDNA. 3’ Poly A tailing was initiated by addition of 4 µL of 10 × TdT buffer and 4 µL of 2.5 mM CoCl2 and water to 9 µL of cDNA in a final volume of 33 µL, denatured at 95 °C for 5 min, and snap cooled. Then 2 µL of 1 mM dATP and 0.5 µL of 20 U/µL Terminal Transferase (#M0315, New England Biolabs, Ipswich, MA, USA) were added and the solution was incubated for 10 min at 37 °C. Finally, 2 µL of 1 mM ddATP was spiked into the reaction and it was incubated at 37 °C for ½ h, and 70 °C for 10 min to inactivate the enzyme.

### 4.13. RNA Sequencing Analysis

The *Enterococcous caceae* gene sequences were downloaded from the National Center for Biotechnology Information (NCBI, Bethesda, MD, USA) under accession number NZ_KB946335. Then UCLUST [64] with default settings and an identity of 90% was used to cluster all the reads from the 4 helicos samples against reference genes from the *E. caceae*. Gene expression was measured by reads per kilobase of mapped reads (RPKM [67]), where the number of reads mapped to the gene was normalized to the length of the genes and the total number of mapped reads to all the genes. Because each sample contains two replicates, differentially expressed genes were identified using the Fisher’s exact test implemented in the edgeR [68] package with replicates. This method fits a negative binomial distribution by estimating the sample dispersion from the replicates and identifies differentially expressed genes that show statistical significance in fold change.

### 4.14. Short Chain Fatty Acid Quantification by GC-MS

Samples were harvested from each bioreactor at 4, 24, and 48 h post inoculation. Samples were centrifuged (Sorvall Legend XTR, ThermoFisher, Waltham, MA, USA) at 5000 rpm for 10 min. The supernatant was drawn into a syringe and sterile filtered using a 0.22 PES (Corning) syringe filter and transferred new 5 mL vials. The fluid was then stored in −80 °C freezer until analysis.

To begin SCFA quantification, the samples were thawed at 40 °C for 30 min. The total SCFAs were extracted from the media with diethyl ether for GCMS (QP2010 Ultra, Shimadzu Scientific, Columbia, MD, USA) analysis. The GC-MS method involved injecting 1 µL of sample into the 260 °C injection port. Using a split ratio of 1:20 and a flow rate of 1.00 mL/min of helium, the sample was deposited on the Stabilwax-DA 30 m × 0.25 mm column (Restek Corporation, Bellefonte, PA, USA) which was held at 125 °C for 1 min and then ramped to 170 °C at 30 °C/min, then to 220 °C at 10 °C/min and then to 250 °C at 50 °C/min where the temperature was held for 3 min. The interface temperature between the GC and MS was held at 250 °C and the ion source temperature was 220 °C.

For each time point, a 2-tailed, unpaired homoscedastic Student’s *t*-test was run to determine if the concentration difference between the control and the experimental groups was statistically significant, *p* < 0.05.

## 5. Conclusions

For the first time, the effect of apigenin on the human gut microbiota was documented. The results of testing using a reductionist model demonstrated that this effect varied with species. Among the species tested, the most effective inhibition by apigenin was observed on *E. caccae*. Analysis of RNA expression indicated that apigenin affects *E. caccae* cell wall/membrane synthesis and increased the incidence of protein misfolding. *B. galacturonicus* growth was also inhibited by apigenin, while *B. catenulatum* and *L. rhamnosus* GG were not affected. In the bacterial community cultured in vitro, apigenin promoted overall growth and diversity, lowered Firmicutes to Bacteroidetes ratio, and promoted production of short chain fatty acids, including butyrate, which is associated with health. Taken together, these observations may explain the health benefits of an apigenin-rich diet.

## Figures and Tables

**Table 1:** RNA expression profiles.

(A) *Enterococcus caccae* Genes Upregulated in Response to Apigenin			
Gene	Description	Fold ▲	Description of Function
*UC7_RS11115*	hypothetical protein	7.0	Unknown
*UC7_RS15590*	hypothetical protein	5.9	Unknown
*UC7_RS11110*	glycerophosphoryl diester phosphodiesterase	2.9	Phospholipid metabolism
*UC7_RS11955*	tyrosine recombinase XerC	2.8	DNA dimer resolution
*UC7_RS11545*	hypothetical protein	2.7	Unknown
*UC7_RS16330*	acyl carrier protein	2.6	Fatty acid biosynthesis
*UC7_RS16340*	MarR family transcriptional regulator	2.5	Stress response
*UC7_RS13900*	glycine cleavage system protein H	2.4	Glycine degradation
*UC7_RS11945*	ATP-dependent protease ATPase subunit HslU	2.3	Protein quality control
*UC7_RS16335*	3-oxoacyl-ACP synthase III	2.3	Type II fatty acid synthesis
*UC7_RS13125*	hypothetical protein	2.2	Unknown
*UC7_RS11950*	ATP-dependent protease subunit HslV	2.2	Protein quality control
*UC7_RS14535*	chaperonin	2.2	Protein folding
*UC7_RS11290*	phosphoglycerol transferase	2.1	Lipoteichoic acid biosynthesis
*UC7_RS15845*	hypothetical protein	2.1	Unknown
*UC7_RS13905*	spx/MgsR family transcriptional regulator	2.1	Stress response
*UC7_RS11940*	GTP-sensing transcriptional pleiotropic repressor CodY	2.1	Transcriptional regulation
*UC7_RS13910*	cell cycle protein FtsW	2.1	Cell division
*UC7_RS12345*	CBS domain-containing protein	2.1	metabolism
*UC7_RS13095*	N5-carboxyaminoimidazole ribonucleotide mutase	2.1	Purine synthesis
*UC7_RS15130*	acyltransferase	2.1	Peptidoglycan synthesis
*UC7_RS12825*	phenazine biosynthesis protein PhzF	2.0	Phenazine biosynthesis
*UC7_RS13565*	hypothetical protein	2.0	Unknown
*UC7_RS15200*	excinuclease ABC subunit B	2.0	DNA repair
*UC7_RS13270*	neutral zinc metallopeptidase	1.9	Extracellular metabolism
*UC7_RS14315*	magnesium-transporting ATPase	1.9	Magnesium transportation
*UC7_RS13560*	diaminopimelate dehydrogenase	1.8	Lysine biosynthesis
*UC7_RS11285*	hypothetical protein	1.8	Unknown
*UC7_RS12620*	undecaprenyl pyrophosphate synthase	1.8	Peptidoglycan biosynthesis
*UC7_RS15260*	hypothetical protein	1.7	Unknown
*UC7_RS16325*	enoyl-ACP reductase II	1.7	Fatty acid biosynthesis
*UC7_RS12170*	hypothetical protein	1.7	Unknown
*UC7_RS11935*	aldolase 1 epimerase LacX	1.7	Carbohydrate metabolism
*UC7_RS11340*	cold-shock protein	1.7	Stress response
*UC7_RS15195*	UvrABC system protein A	1.7	DNA repair
*UC7_RS13110*	hypothetical protein	1.7	Unknown
*UC7_RS13815*	hypothetical protein	1.7	Unknown
*UC7_RS13990*	hypothetical protein	1.7	Unknown
*UC7_RS13115*	*N*-acetylmuramoyl-l-alanine amidase	1.7	Cell separation
*UC7_RS16345*	hypothetical protein	1.7	Unknown
*UC7_RS12340*	2_3_4_5-tetrahydropyridine-2_6-dicarboxylate *N*-acetyltransferase	1.7	Lysine biosynthesis
*UC7_RS13365*	NrdR family transcriptional regulator	1.7	DNA synthesis and repair
*UC7_RS14530*	molecular chaperone GroEL	1.7	Protein folding
*UC7_RS14300*	GNAT family acetyltransferase	1.6	Transcriptional regulation
*UC7_RS16190*	penicillin-binding protein 2B	1.6	Cell wall synthesis
*UC7_RS13090*	phosphoribosylaminoimidazole carboxylase ATPase subunit	1.6	Purine and pyrimidine ribonucleotide biosynthesis
*UC7_RS15255*	hypothetical protein	1.6	Unknown
*UC7_RS13120*	hypothetical protein	1.6	Unknown
*UC7_RS13335*	chaperone protein ClpB	1.6	Protein folding
*UC7_RS14685*	DNA-binding response regulator	1.6	Transcriptional regulation
*UC7_RS11985*	S26 family signal peptidase	1.6	Protein maturation
*UC7_RS12190*	elongation factor Tu	1.6	Protein synthesis
*UC7_RS15900*	d-alanine-poly(phosphoribitol) ligase subunit 2	1.6	Cell wall/membrane metabolism
*UC7_RS16320*	malonyl CoA-ACP transacylase	1.5	Fatty acid and polyketide synthesis
*UC7_RS13160*	penicillin-binding protein	1.5	Cell wall synthesis
*UC7_RS15715*	50S ribosomal protein L10	1.5	Protein translation
*UC7_RS15600*	phosphocarrier protein HPr	1.5	Carbohydrate phosphorylation
*UC7_RS13265*	hypothetical protein	1.5	Unknown
*UC7_RS15250*	hypothetical protein	1.5	Unknown
*UC7_RS12410*	signal peptidase I	1.5	Protein maturation
**(B) *Enterococcus caccae* Genes Downregulated in Response to Apigenin**			
**Gene**	**Description**	**Fold ▼**	**Description of Function**
*UC7_RS16160*	hypothetical protein	2.4	Unknown
*UC7_RS16570*	hypothetical protein	2.2	Unknown
*UC7_RS14780*	hypothetical protein	2.0	Unknown
*UC7_RS13490*	Lsa family ABC-F type ribosomal protection protein	2.0	Ribosomal protection
*UC7_RS14785*	hypothetical protein	1.9	Unknown
*UC7_RS11535*	hypothetical protein	1.9	Unknown
*UC7_RS15700*	general stress protein GlsB	1.9	Stress response
*UC7_RS14775*	hypothetical protein	1.8	Unknown
*UC7_RS15645*	hypothetical protein	1.8	Unknown
*UC7_RS16575*	hypothetical protein	1.8	Unknown
*UC7_RS16225*	WxL domain surface protein	1.7	Surface protein binding
*UC7_RS13920*	hypothetical protein	1.7	Unknown
*UC7_RS15555*	MocD family protein	1.7	Catabolism of mannopine
*UC7_RS13450*	hypothetical protein	1.7	Unknown
*UC7_RS16165*	hypothetical protein	1.7	Unknown
*UC7_RS13865*	SufS family cysteine desulfurase	1.6	Fe-S protein biosynthesis
*UC7_RS13860*	iron-sulfur cluster assembly scaffold protein	1.6	Iron-sulfur cluster formation
*UC7_RS14795*	deoxycytidine triphosphate deaminase	1.6	Nucleotide metabolism
*UC7_RS13855*	FeS assembly protein SufB	1.6	Iron-sulfur cluster formation
*UC7_RS13455*	hypothetical protein	1.6	Unknown
*UC7_RS11040*	hypothetical protein	1.6	Unknown
*UC7_RS12130*	hypothetical protein	1.6	Unknown
*UC7_RS13465*	hypothetical protein	1.5	Unknown
*UC7_RS12125*	hypothetical protein	1.5	Unknown
*UC7_RS14270*	ATP synthase subunit C	1.5	ATP synthesis
*UC7_RS16230*	hypothetical protein	1.5	Unknown
*UC7_RS16220*	hypothetical protein	1.5	Unknown
*UC7_RS11885*	cardiolipin synthase	1.5	Phospholipid biosynthesis
*UC7_RS14805*	ATP-grasp domain-containing protein	1.5	metabolism
*UC7_RS15830*	thioredoxin-disulfide reductase	1.5	Redox regulation
